# ATP Triggers Human Th9 Cell Differentiation via Nitric Oxide-Mediated mTOR-HIF1α Pathway

**DOI:** 10.3389/fimmu.2019.01120

**Published:** 2019-05-20

**Authors:** Suyasha Roy, Amit Awasthi

**Affiliations:** Immuno-Biology Lab, Translational Health Science and Technology Institute, Faridabad, India

**Keywords:** T helper cell (Th), inflammation, T helper 9 cell, cytokines, transcription activation

## Abstract

Interleukin 9 (IL-9)-producing helper T (Th9) cells have a crucial effector function in inducing allergic inflammation, autoimmunity, immunity to extracellular pathogens and anti-tumor immune responses. Although the cytokines that lead to the differentiation of human Th9 cells have been identified, other factors that support the differentiation of Th9 cells have not been identified yet. Here we show that the extracellular ATP (eATP) induces the differentiation of Th9 cells. We further show that eATP induces the production of nitric oxide (NO), which create a feed forward loop in the differentiation of human Th9 cells, as inhibition of purinergic receptor signaling suppressed the generation of human Th9 cells while exogenous NO could rescue generation of Th9 cells even upon inhibition of purinergic receptor signaling. Moreover, we show that ATP promotes mTOR and HIF1α dependent generation of Th9 cells. Our findings thus identify that ATP induced nitric oxide potentiate HIF1α-mediated metabolic pathway that leads to IL-9 induction in Th9 cells. Here we identified that the ATP-NO-mTOR-HIF1α axis is essential for the generation of human Th9 cells and modulation of this axis may lead to therapeutic intervention of Th9-associated disease conditions.

## Introduction

Interleukin (IL) 9, a pleotropic cytokine, was initially described as T cell growth factor, as it signals through common gamma chain on T cells (1, 2). In addition to T cells, the role of IL-9 was also demonstrated as mast cell growth factor (3). IL-9 functions through IL-9 receptor consisting of IL-9 receptor α chain and the γ chain (4). Upon binding to IL-9 receptor, IL-9 induces effector functions and activates mast cells, eosinophils during allergic inflammation (5, 6). The most precise role of IL-9 was found to be associated with human allergic inflammation, both IL-9 and IL-9R was found to be genetically linked to human asthma (7). Consistently, subsequent studies have shown that neutralization of IL-9 with anti-IL-9 antibody reduced the allergic inflammation in animal model of asthma, suggesting an important role of IL-9 in disease pathogenesis (7). Consistently, pulmonary overexpression of IL-9 was found to enhance immunopathology in allergic inflammation in asthma in mice (8).

Initial studies have identified IL-9 as Th2 cytokine, as *in vivo* neutralization of IL-4 substantially blocked the production of IL-9 during *Leishmania* infection (9). Most of the initial studies on IL-9 were conducted in Th2-biased Balb/c animal models, and therefore it was suggested that IL-9 enhance Th2-associated disease pathogenesis in *Leishmania* infection as well as allergic inflammation in asthma. Based on these studies, it was clearly established that IL-9 is primarily produced by T cells, its production is found to be increased with the expansion of Th2 cells. The clarity of IL-9 induction in T cells came up with the identification of a T cell population, which predominantly produce IL-9 without expressing lineage-specific cytokines of Th1, Th2 and Th17 cells (10, 11).

The identification of differentiation factors of Th9 cells led to reconcile the association of IL-9 with Th2 cells, as IL-4 is one of the Th2 cytokines required in combination with TGF-β1 to induce the developmental program for Th9 cells (10, 11). The developmental pathway of Th9 cells and iTregs is reciprocally regulated. While TGF-β1 induces the expression of Foxp3, IL-4 not only suppresses the TGF-β1-induced expression of Foxp3 but together with TGF-β1 induces IL-9-producing Th9 cells. Similar to murine Th9 cells, TGF-β1, and IL-4 were also found to induce the differentiation of human Th9 cells (10, 12).

Since IL-9 is primarily associated in allergic inflammation, the functions of Th9 cells was found to be associated in allergic diseases. In addition, Th9 cells are also crucial for the pathogenesis of IBD, EAE and anti-tumor immunity. In recent studies, using the mice model of cancer, the anti-tumor functions of Th9 cells were described (13, 14). It was shown that IL-21 and IL-1β enhance the anti-tumor functions of Th9 cells in IFN-γ dependent manner (15, 16). Subsequent studies have shown that Th9 cells could suppress the tumor growth in antigen-specific and antigen non-specific manner. In addition, it was recently demonstrated that GMCSF and TNF-α profoundly enhanced the anti-tumor functions of Th9 cells (17, 18). Moreover, the role of Th9 cells in eradicating advance tumor were also identified and suggested that IL-9, Eomes, and Traf6 of Th9 cells are essential in eradicating advance tumor (19).

Although the differentiating cytokines that lead to Th9 cell induction were identified, other environmental cues like nutrients, local oxygen levels, and metabolites are yet to be defined. Here we report that the extracellular ATP (eATP) promotes human Th9 cell differentiation, as blocking of purinergic receptor signaling suppressed the generation of Th9 cells. We further show that eATP induces the production of nitric oxide (NO) and creates a feed-forward loop to potentiate the human Th9 cells differentiation. However, ATP and NO independently activate mTOR-HIF-1α pathway, which in turn support Th9 cell differentiation. ATP triggers NO-mediated mTOR-HIF-1α signaling, which increases glycolysis and IL-9 production in human Th9 cells. Our findings identify for the first time that ATP-induced NO potentiates mTOR-HIF-1α-mediated metabolic signaling pathway that is required for IL-9 induction in Th9 cells, thus may lead to therapeutic intervention of Th9-associated disease conditions.

## Materials and Methods

### Human T Cell Culture

Human Th9 cells differentiation is induced as described earlier (12, 20). All human experiments were performed in accordance to the approved guidelines of Human Ethics Committee of THSTI. Human blood samples were collected from healthy individuals after the written informed consent. Healthy individuals were enrolled in this study based on the inclusion/exclusion criteria prescribed by the Human Ethics Committee of THSTI. Naïve T cells (CD4^+^CD25^−^CD45RA^+^) were sorted by FACS Aria III (BD Bioscience) with ~95% purity from PBMCs isolated from healthy donors using Ficoll-Paque Plus (GE Healthcare) gradient centrifugation. Sorted naïve CD4^+^ T cells (0.1 × 10^6^)/well were stimulated with plate-bound anti-hCD3 (10 μg/ml; UCHT1, Bio X cell) and soluble anti-hCD28 (2 μg/ml; 28.2, Bio X cell) in round-bottom 96-well plate. The cells were cultured for 6 days in the presence of IL-12 (10 ng/ml) for Th1, IL-4 (10 ng/ml) for Th2, TGF-β1 (5.0 ng/ml) and IL-4 (10 ng/ml) for Th9, TGF-β1 (5.0 ng/ml), IL-1β (12.5 ng/ml), IL-6 (25ng/ml), IL-21 (25 ng/ml) and IL-23 (25ng/ml) for Th17, TGF-β1 (5ng/ml) and IL-2 (50 U/ml) for Treg cell differentiation (12, 21).

Hypoxia experiments were carried out in a hypoxia chamber (Coy Laboratory Products, Grass Lake, MI, USA) where cells were subjected to 1% oxygen at 37°C in 5% CO_2_. For pharmacological chemical treatments, cells were incubated with 1 mM 2-DG (Sigma Aldrich, St. Louis, MO, USA); 50 nM rapamycin (Sigma Aldrich); 5 μM acriflavine (Sigma Aldrich); 0.5 mM L-NIL (NO inhibitor) (Sigma Aldrich)**;** 30 μM Suramin (Sigma Aldrich), 100 μM NOC-18 (NO donor) (Sigma Aldrich), 50 μM ATP (Sigma Aldrich) at the onset of culture.

### Lentiviral Transduction

For transduction, the lentiviral plasmid was packed as described earlier using HEK293T packaging cell line with X-tremeGENE 9 Transfection Reagent (Roche, Germany). Naïve T cells (0.1 × 10^6^)/well were transduced with lentivirus upon activation with plate-bound anti-hCD3 (10 μg/ml; UCHT1, Bio X cell) and soluble anti-hCD28 (2 μg/ml; 28.2, Bio X cell). The virus-containing media was replaced with fresh medium containing TGF-β1 (5.0 ng/ml) and IL-4 (10 ng/ml) for Th9 differentiation and cultured for 6 days at 37°C.

### Nucleofection of Human T Cells

Nucleofection of naïve human CD4^+^ T cells was performed as described earlier (20). Naïve T cells (1.5 × 10^6^) were resuspended in 100 μl Amaxa™ 4D-Nucleofector™ Solution and nucleofected with PBS/pU6-HIF1 alpha RNAi plasmid 2 (Addgene, Plasmid #21104) on 4D-Nucleofector™ X unit (Lonza, Walkersville, MD, USA) using P3 Primary Cell 4D-Nucleofector™ X Kit (Lonza, Walkersville, MD, USA) as per manufacturer's protocol.

### Flow Cytometry

For intracellular cytokine staining, cells were restimulated on day 7 post-culture with Phorbol 12-myristate 13-acetate (PMA; 50 ng/ml; Sigma-Aldrich), ionomycin (500 ng/ml; Sigma-Aldrich), and Golgi stop (BD Biosciences) for 5 h at 37°C. Intracellular and surface staining was performed as described earlier. Data were acquired on a FACSVerse (BD Biosciences) and analyzed using FlowJo software (Tree Star, Ashland, OR, USA) (10, 12).

### qPCR

mRNA was extracted from cells after *in vitro* differentiation using the RNeasy Mini Kit (Qiagen, Venlo, The Netherlands) and converted to cDNA using iScript cDNA Synthesis Kit (Bio-Rad Laboratories, Hercules, CA, USA). qPCR was performed with the SYBR Green Gene Expression Assay using the ABI Fast 7500 Dx qPCR system (Applied Biosystems) according to the manufacturer's protocol (10). The target gene expression was normalized to GAPDH, and the fold change was calculated as 2^−ΔΔ*CT*^ comparative threshold. Briefly qPCR results were analyzed with SDS2.1 software. The cycling threshold value of the endogenous control (*gapdh/bactin*) was subtracted from the cycling threshold value of the target gene to generate the change in the cycling threshold (ΔCT). The relative expression of each target gene is expressed as the “fold change.” We used this previously used formula (POWER(2, -ΔCT)^*^10,000 to calculate the relative expression of gene (20).

The following primers sets were used: *GAPDH* (forward, 5′-ACAGTTGCATGTAGACT-3′; reverse, 5′-TTTTTGGTTGAGCACAGG-3′), *Il9* (forward, 5′-GACATCAACTTCCTCATC-3′; reverse, 5′-GAGACAACTGGTCTTCTGG-3′), *HIF1*α (forward, 5′-AAAATCTCSTCCSSGAAGCC-3′; reverse, 5′-AATGTTCCAATTCCTACTGC-3′), *Nos2* (forward, 5′-AGCTCAACAACAAATTCAGG-3′; reverse, 5′-ATCAATGTCATGAGCAAAGG-3′), *mTOR* (forward, 5′-AGCAGAGAAAGGTTTTGATG-3′; reverse, 5′-GATCTCCTCCATCTCTTCTC-3′), *IRF4* (forward, 5′-TGACTCTATGCTTTGGAGAG-3′; reverse, 5′-GCTAAACTCCTAAGTACGTG-3′). All the primer sets were purchased from Sigma-Aldrich.

### Enzyme-Linked Immunosorbent Assay (ELISA)

The concentration of human IL-9 was measured by ELISA carried out with paired antibodies according to manufacturer's instructions (BioLegend). The plates were analyzed on a Synergy™ HT Multi-Detection Microplate Reader, BioTek (Winooski, VT, USA) (10).

### Nitrite Determination

The nitric oxide production was estimated in cell culture supernatants by measuring the nitrite concentration, a stable NO product, using Griess Reaction. 50 μl of each experimental sample and 100 μl of Griess reagent (0.1% naphthylethylenediamine dihydrochloride and 1% sulphanilamide in 2% phosphoric acid) was added in duplicates in 96-well microtiter plates (22). After incubating for 10 min in dark, absorbance was read at 540 nm using ELISA Plate Reader (Bio-Rad Laboratories).

### Lactate Assay

Accumulation of lactate, the end-product of glycolysis, were determined in cell culture supernatants harvested on day 7 using the Lactate Colorimetric Assay Kit II (Sigma-Aldrich, MAKO65, USA) according to the manufacturer's protocol.

### ATP Determination

The amount of ATP secreted in the cell culture supernatants harvested on day 7 post-culture was determined by ATP Determination Kit (Molecular Probes, invitrogen detection technologies; A22066, USA) according to manufacturer's instructions as described earlier (23).

### Statistical Analysis

All the statistical analysis was done using GraphPad Prism 7.0 software (La Jolla, CA, USA). Two-tailed Student's *t-*test were used for comparison of means between two groups and one-way ANOVA test followed by Tukey's multiple comparison's test was used for comparison of means between more than two groups. Multiple groups with two variables were compared using two-way ANOVA followed by Tukey's multiple comparison's test. All the data are presented as mean ± SEM. *P* < 0.05 were considered statistically significant for all the experiments.

## Results

### ATP Enhances Human Th9 Cell Differentiation

**Figure 1:**
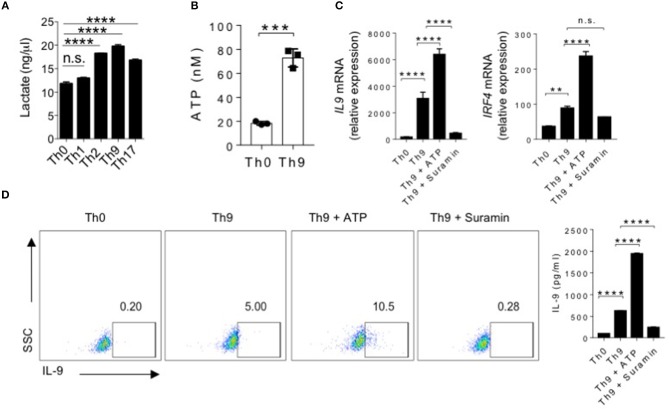
ATP enhances human Th9 cell differentiation. **(A)** Lactate Assay showing accumulation of lactate in cell culture supernatants after 6 days of culture under Th0, Th1, Th2, Th9, and Th17 inducing conditions. **(B)** Extracellular ATP in culture supernatants of Th0 and Th9 cells. **(C)** Sorted naïve T cells differentiated under Th0 and Th9 polarizing conditions for 6 days in the presence of ATP and Suramin followed by examination of mRNA expression of IL-9 and IRF4. **(D)** Intracellular cytokine staining for IL-9 and IL-9 production in the culture supernatants estimated by ELISA. Data are representative of mean ± SEM from three independent experiments (*n* = 3). ^*^P < 0.0332, ^**^P < 0.0021, ^***^P < 0.0002, ^****^P < 0.0001; one-way ANOVA followed by Tukey's test **(A,C,D)**, Student's *t*-test **(B)**.

T cells activation and differentiation relies on aerobic glycolysis, instead of oxidative phosphorylation, in order to meet their increased biosynthetic energy demands (24–26). As a result of glycolysis and metabolic activities, lactate accumulate upon T cell activation (24, 27, 28). To understand the metabolic requirements during human Th9 cells differentiation, we measured the lactate production in effector subsets of human Th cells. Interestingly, our data indicates that human Th9 cells produce substantially higher levels of lactate as compared to other Th subsets (Figure 1A). In fact, Th2, Th9, and Th17 cells, which produce IL-9, also produce higher amounts of lactate as compared to non-IL-9-producing Th0 and Th1 cells (Figure 1A). Since glycolysis leads to lactate production coupled with ATP generation (24, 29, 30), therefore we tested whether Th9 cells also generate higher levels of ATP to support the energy demands of Th9 cells. As compared to Th0, Th9 cells generate significantly higher amounts of ATP (Figure 1B), which led us to hypothesize whether ATP contributes to the differentiation of human Th9 cells. Although the role of eATP has been established in Th17 and Tregs cells differentiation and functions (23, 31, 32), the contribution of ATP in the differentiation of human Th9 cells is not identified yet. To understand the role of ATP in the differentiation of human Th9 cells, we supplemented Th9 culture condition with eATP. Our data indicate that the supplementation of eATP in the presence of TGF-β1 and IL-4 further enhanced IL-9 production in Th9 cells (Figure 1C). To further substantiate our claim, we blocked the P2X and P2Y P_2_-purinergic receptors using suramin to decipher whether blocking the binding of eATP affects IL-9 induction in human Th9 cells. Consistently, suramin inhibited *Il9* expression and IL-9 production in human Th9 cells (Figures 1C,D). Since IRF4 is crucial for the induction and differentiation of Th9 cells (33), therefore we tested whether eATP enhances the expression of IRF4 in human Th9 cells. Our data clearly indicate that eATP enhances the expression of IRF4 in human Th9 cells (Figure 1C), suggesting that eATP contribute to enhanced differentiation of human Th9 cells. Taken together these data collectively indicates that ATP is essential for enhancing IL-9 induction in Th9 cells.

### ATP Is Essential for NO-Mediated Induction of IL-9 in Human Th9 Cells

**Figure 2:**
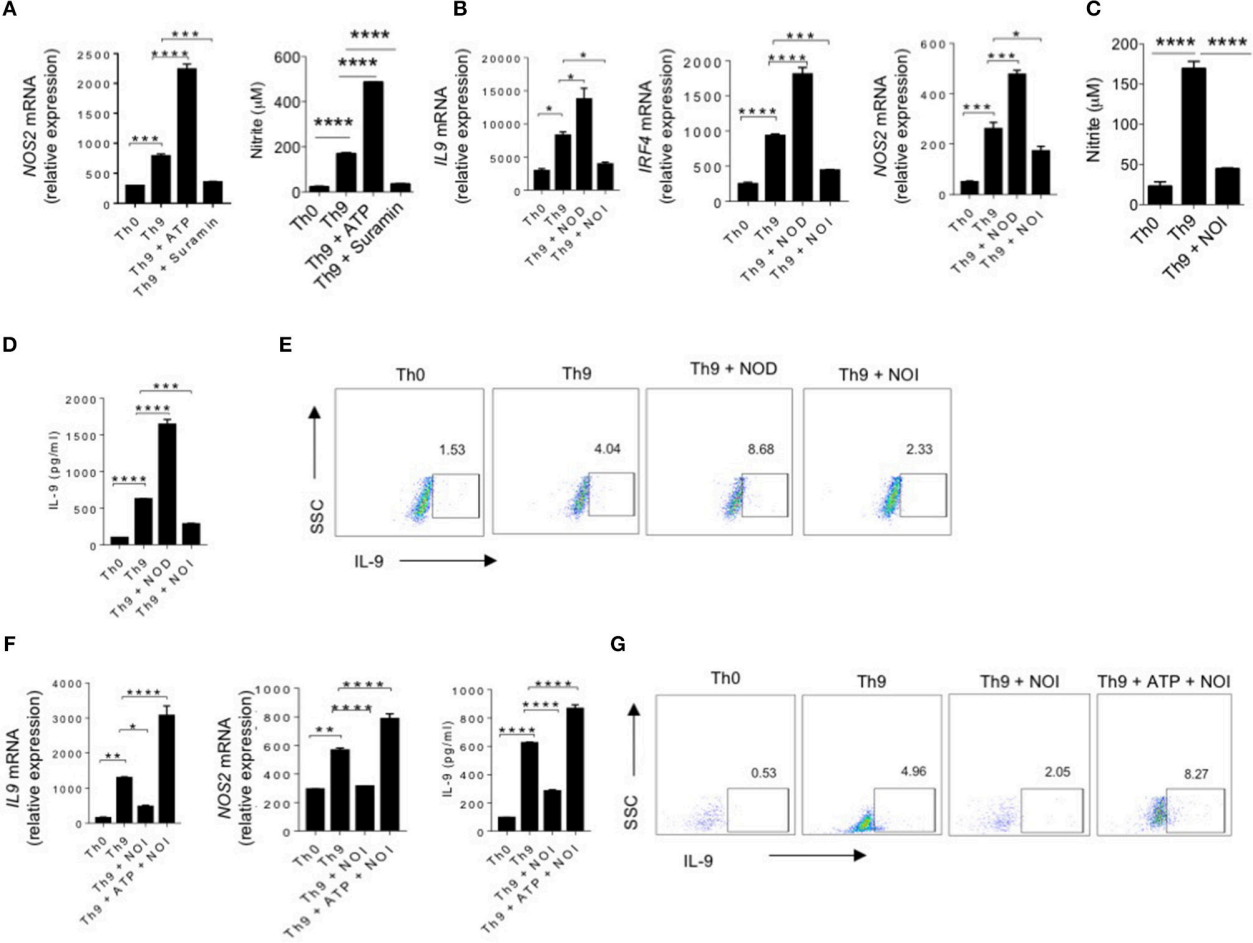
ATP is essential for NO-mediated induction of IL-9 in human Th9 cells. **(A)** Sorted naïve T cells differentiated under Th0 and Th9 polarizing conditions for 6 days in the presence of ATP and Suramin followed by examination of mRNA expression of NOS2 and nitrite production in the culture supernatants. **(B)** Sorted naïve T cells differentiated under Th0 and Th9 polarizing conditions for 6 days in the presence of NOD (NO donor) and NOI (NO inhibitor) followed by examination of mRNA expression of IL-9, IRF4 and NOS2. **(C)** Nitrite measurement in the culture supernatants. **(D)** IL-9 production in the culture supernatants estimated by ELISA **(E)** Intracellular cytokine staining for IL-9 production. **(F)** Sorted naïve T cells differentiated under Th0 and Th9 polarizing conditions for 6 days in the presence of NOI (NO inhibitor) and ATP+NOI followed by examination of mRNA expression of IL-9 and NOS2 and ELISA estimation for IL-9 production. **(G)** Intracellular cytokine staining for IL-9. Data are representative of mean ± SEM from three independent experiments (*n* = 3). ^*^P < 0.0332, ^**^P < 0.0021, ^***^P < 0.0002, ^****^P < 0.0001; one-way ANOVA followed by Tukey's test **(A–D, F)**.

The role of NO was tested in T cells differentiation, as NO was found to suppress IL-17 while it promotes IL-9 in Th17 cells (22, 34). Furthermore, the role of NO was also found to directly influence IL-9 induction in Th9 cells, as NOS2 deficiency was found to attenuate IL-9 induction and differentiation of Th9 cells (22, 24). It was shown that ATP contribute to the generation of NO production (35). Based on this observation, we wanted to test whether ATP promotes induction of IL-9 in human Th9 cells by potentiating NO production. To do this, we first tested whether eATP enhances the generation of NO in human Th9 cells. Differentiation of human Th9 cells in the presence of eATP has found to increase the expression of NOS2 and production of NO in Th9 cells (Figure 2A). Consistently, blocking of P2X and P2Y P_2_-purinergic receptors by suramin suppressed the expression of NOS2 and NO production in human Th9 cells, indicating a possibility that ATP-mediated NO might be essential for enhanced IL-9 production in human Th9 cells. Interestingly, we found that supplementation of NO in Th9 cell cultures enhances IL-9 production in human Th9 cells (Figures 2B–E). Contrary to NO supplementation, NO inhibition suppressed IL-9 induction in human Th9 cells (Figures 2B-E). We further tested whether ATP can rescue the Th9 cells differentiation in the absence of NO. Our data indicate that eATP was able to rescue IL-9 production in the absence of NO generation in human Th9 cells (Figures 2F,G). Taken together, it implies that ATP-NO creates a feed forward loop that is essential for the production of IL-9 and Th9 cells differentiation.

### ATP-mTOR Pathway Induce Human Th9 Cells Differentiation

**Figure 3:**
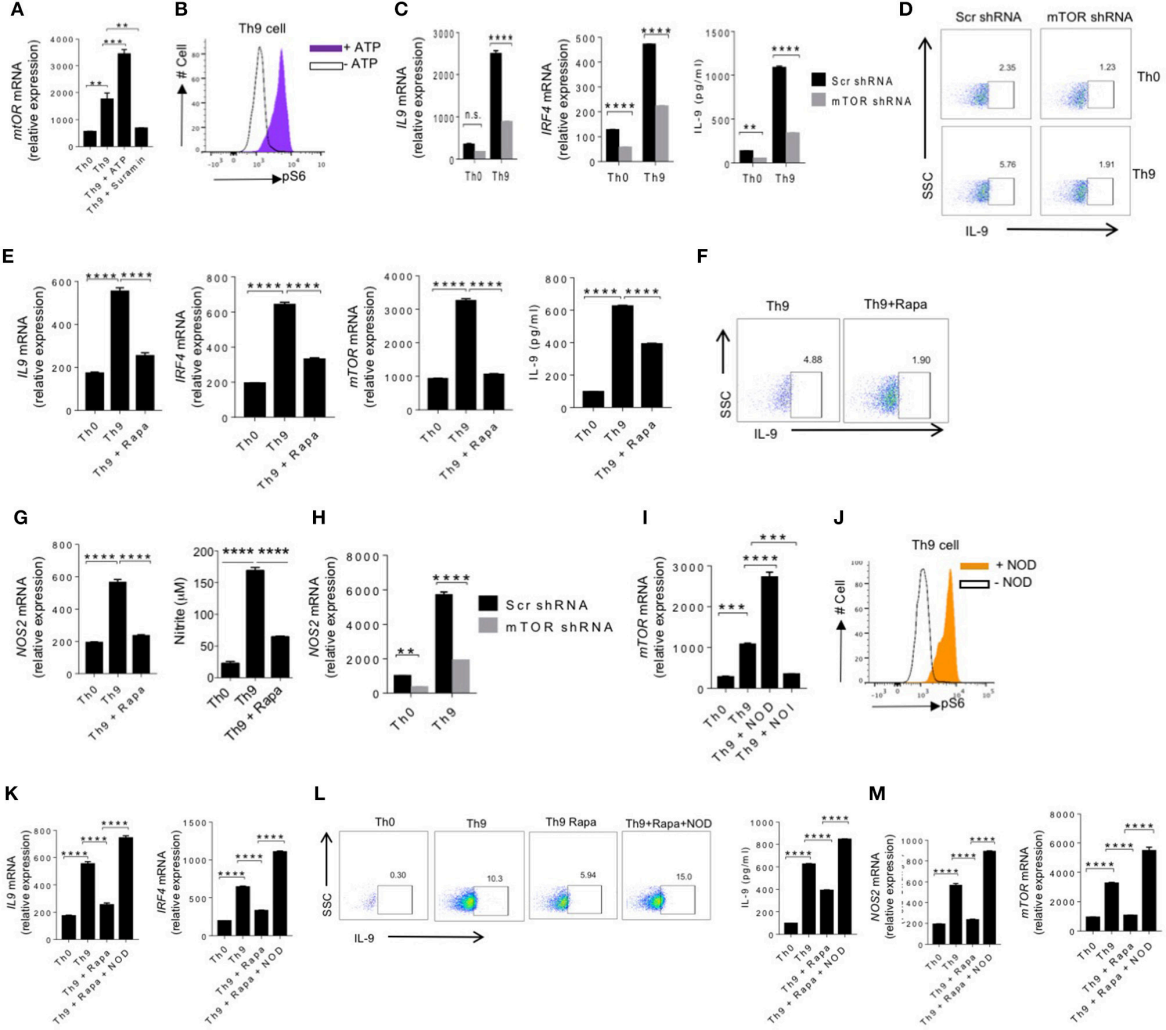
ATP-mTOR pathway induce human Th9 cells differentiation. **(A)** Sorted naïve T cells were differentiated under Th0 and Th9 polarizing conditions for 6 days in the presence of ATP and Suramin followed by examination of mRNA expression of mTOR. **(B)** intracellular cytokine staining of the phosphorylation of S6 in the presence and absence of ATP in Th9 cells. **(C)** Sorted naïve T cells were activated with anti-CD3/CD28 for 24 h and differentiated under Th0 and Th9 skewing conditions, and transduced with control lentivirus carrying scramble shRNA or mTOR shRNA-expressing lentivirus followed by examination of mRNA expression of IL-9 and IRF4 and ELISA for IL-9 production. **(D)** Intracellular cytokine staining for IL-9 production. **(E)** Sorted naïve T cells were differentiated under Th0 and Th9 polarizing conditions for 6 days in the presence and absence of RAPA (rapamycin) followed by examination of mRNA expression of IL-9, IRF4 and mTOR and ELISA for IL-9 production. **(F)** Intracellular cytokine staining for IL-9 production. **(G)** Sorted naïve T cells were differentiated under Th0 and Th9 polarizing conditions for 6 days in the presence and absence of RAPA (rapamycin) followed by examination of mRNA expression of NOS2 and nitrite measurement in the culture supernatants. **(H)** Sorted naïve T cells were activated with anti-CD3/CD28 for 24 h and polarized under Th0 and Th9 skewing conditions, and transduced with control lentivirus carrying scramble shRNA or mTOR shRNA-expressing lentivirus followed by examination of mRNA expression of NOS2. **(I)** Sorted naïve T cells were differentiated under Th0 and Th9 polarizing conditions for 6 days in the presence of NOD and NOI (NOD-NO donor; NOI-NO inhibitor) followed by examination of mRNA expression of mTOR. **(J)** Intracellular cytokine staining of the phosphorylation of S6 in the presence and absence of NOD in Th9 cells. **(K)** Sorted naïve T cells were differentiated under Th0 and Th9 polarizing conditions for 6 days alone, in the presence of RAPA (Rapamycin) and RAPA+NOD followed by examination of mRNA expression of IL-9 and IRF4. **(L)** Intracellular cytokine production of IL-9 and estimation of IL-9 production in the culture supernatants by ELISA. **(M)** Examination of mRNA expression of NOS2 and mTOR. Data are representative of mean ± SEM from three independent experiments (*n* = 3). ^*^P < 0.0332, ^**^P < 0.0021, ^***^P < 0.0002, ^****^P < 0.0001; one-way ANOVA followed by Tukey's test **(A,E,G,I,K,M)**, two-way ANOVA followed by Tukey's test **(C,H)**.

To understand the molecular mechanisms of contribution of eATP in enhancing human Th9 cells differentiation, we hypothesized to test whether ATP-activated mTOR pathway plays a role in the differentiation of human Th9 cells, as it was shown that ATP activates mTOR pathway (36, 37). To test our hypothesis, we differentiated human naïve CD4^+^ T cells into Th9 cells in the presence of eATP. As shown earlier, while eATP increased IL-9 production, it also enhanced mTOR and pS6 kinase expression, a downstream molecule in mTOR pathway, in human Th9 cells (Figures 3A,B). Next we tested whether mTOR is required for the differentiation of human Th9 cells. To do this, we suppressed mTOR using mTOR shRNA during human Th9 cells differentiation. Interestingly, mTOR inhibition significantly blocked human Th9 cells differentiation and IL-9 generation, as determined by IL-9 production and IRF4 expression (Figures 3C,D), suggesting that ATP-mediated activation of mTOR is essential in differentiation of human Th9 cells. Consistently, similar to mTOR shRNA inhibition, rapamycin, a pharmacological inhibitor of mTOR, also suppressed mTOR, IRF4 and IL-9 in human Th9 cells (Figures 3E,F). To further understand the role of ATP-NO-mTOR axis in IL-9 production and Th9 cells differentiation, we tested whether ATP-mediated NO is essential for the induction of IL-9 in human Th9 cells through mTOR. We found that rapamycin suppressed the expression of NOS2 and production of NO in Th9 cells (Figure 3G). Since mTOR inhibition shown to suppress glycolysis, therefore we tested the expression of Glut1 and glycolytic genes in the presence of rapamycin. Our data suggests that rapamycin block the expression of Glut1 as well as other glycolytic genes (Supplementary Figure 1). Consistently, we have also found that, similar to rapamycin, mTOR shRNA also blocked the NOS2 expression in human Th9 cells (Figure 3H). To further understand the functional role of NOS2 and NO in the activation of mTOR-mediated Th9 cells differentiation, we tested whether inhibition or supplementation of NO with NOI or NOD, respectively block or enhance mTOR pathway in human Th9 cells differentiation. Our data indicate that inhibition of NO suppressed mTOR expression while supplementation of NO enhanced pS6 Kinase activation in human Th9 cells (Figures 3I,J). As indicated that NO is essential for mTOR activation in Th9 cells, we next tested whether NO can rescue human Th9 cells differentiation in the absence of mTOR activation. Interestingly, supplementation of NO rescued Th9 differentiation as evident by IL-9 and IRF4 induction (Figures 3K,L) by rescuing NOS2 and mTOR expression in human Th9 cells (Figure 3M). These observations together indicate that ATP-NO-mTOR axis is required for human Th9 cells differentiation.

### ATP-Induced HIF-1α Is Required for Human Th9 Cells Differentiation

**Figure 4:**
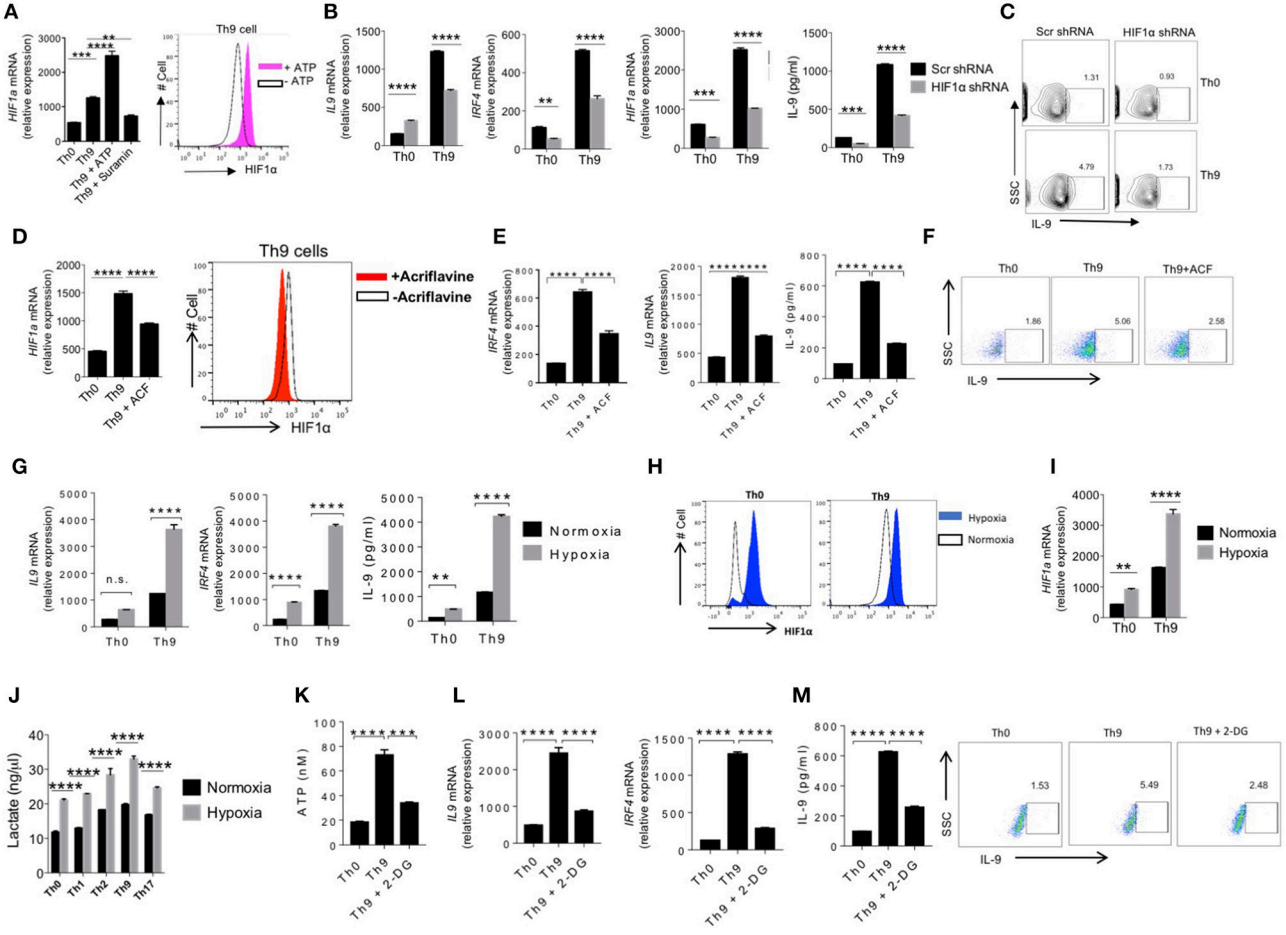
ATP-induced HIF-1α is required for human Th9 cells differentiation. **(A)** Sorted naïve T cells were differentiated under Th0 and Th9 polarizing conditions for 6 days in the presence of ATP and Suramin followed by examination of mRNA expression of HIF1α and intracellular cytokine staining for HIF1α. **(B)** Sorted naïve T cells were nucleofected with naked scramble shRNA and HIF1α shRNA and were differentiated under Th0 and Th9 polarizing conditions for 6 days analyzed for mRNA expression of IL-9, IRF4, HIF1α and IL-9 production in the culture supernatants by ELISA **(C)** Intracellular cytokine staining of IL-9 in Th9 cells. **(D)** Sorted naïve T cells were differentiated under Th0 and Th9 polarizing conditions for 6 days in the presence and absence of acriflavine (ACF) followed by examination of mRNA expression of HIF1α and intracellular cytokine staining for HIF1α. **(E)** mRNA expression of IL-9, IRF4 and IL-9 production in the culture supernatants estimated by ELISA. **(F)** Intracellular cytokine staining for IL-9 expression **(G)** Sorted naïve T cells were differentiated under Th0 and Th9 polarizing conditions for 6 days in normoxia (21% oxygen) and hypoxia (1% oxygen), respectively. Total RNA was extracted, reverse transcribed and real-time PCR was done for analyzing mRNA expression of IL-9 and IRF4 and IL-9 production in the culture supernatants estimated by ELISA. **(H)** Intracellular cytokine staining of HIF1α and **(I)** mRNA expression of HIF1α. **(J)** Sorted naïve T cells differentiated under Th0, Th1, Th2, Th9, and Th17 polarizing conditions for 6 days in normoxia (21% oxygen) and hypoxia (1% oxygen), respectively followed by measurement of lactate production in culture supernatants. **(K)** Sorted naïve T cells were differentiated under Th0 and Th9 polarizing conditions for 6 days in the absence and presence of 2-DG followed by estimation of ATP production in culture supernatants. **(L)** mRNA expression of IL-9 and IRF4. **(M)** Estimation of IL-9 production in culture supernatants by ELISA and intracellular cytokine production of IL-9. Data are representative of mean ± SEM from three independent experiments (*n* = 3). ^*^P < 0.0332, ^**^P < 0.0021, ^***^P < 0.0002, ^****^P < 0.0001; one-way ANOVA followed by Tukey's test **(A,D,E,K–M)**, two-way ANOVA followed by Tukey's test **(B,G,I,J)**.

It is demonstrated that mTOR signaling leads to HIF-1α activation, which play critical role in differentiation and interplay between effector and regulatory T cells (24, 38, 39). Our data indicated that eATP-NO-mTOR axis is essential in production of IL-9 in human Th9 cells. We tested whether ATP promotes IL-9 and differentiation of Th9 cells by inducing HIF-1α. Our data indicated that eATP not only enhanced IL-9 production in Th9 cells (Figure 1) but also increased the expression of HIF-1α at both mRNA and protein level in human Th9 cells (Figure 4A). Consistently, inhibition of eATP signaling by suramin inhibited the expression of HIF-1α in human Th9 cells (Figure 4A). These observations clearly indicated the role of HIF-1α in the differentiation of human Th9 cells. Next we assessed the role of HIF-1α in human Th9 cells differentiation by blocking HIF-1α using shRNA, and found that shRNA-mediated silencing of HIF-1α significantly suppressed IL-9 production as well as the differentiation of human Th9 cells indicated by the attenuated expression of IRF4 and IL-9 (Figures 4B,C). Consistently, we found that acriflavine, which suppresses the hetero-dimerization of HIF-1α and HIF-1β, also suppressed the expression of HIF-1α at mRNA and protein level (Figure 4D). HIF-1α is induced during glycolysis upon T cells activation and differentiation. HIF-1α in turn support glycolysis, as blocking of HIF-1α known to suppress glycolysis. Our data demonstrated that blocking of HIF-1α using acriflavine suppressed the expression of Glut1 together with glycolytic genes (Supplementary Figure 2A). Our data further suggests that acriflavine suppressed both IRF4 and IL-9 expression in Th9 cells (Figures 4E,F), implying the role of HIF-1α in human Th9 cell differentiation. To further support the role of HIF-1α in human Th9 cells differentiation, we found that human Th9 cell differentiation was enhanced under hypoxic condition, as indicated by the enhanced expression of IRF4, IL-9, and increased production of IL-9 together with HIF-1α expression (Figures 4G–I). Our data indicate that Th9 cells, as compared to other Th cells, are highly glycolytic in nature, as they produce an increased amount of lactate (Figure 4J). The lactate production was further enhanced in Th9 cells upon culturing them in hypoxic condition (Figure 4J). In addition, Th9 cells cultured in hypoxic condition were shown to have enhanced expression Glut1 and the genes that are associated with glycolysis (Supplementary Figure 2B). As lactate is generated due to higher consumption of glucose through glycolysis, we tested whether blocking glycolysis using 2-DG suppresses human Th9 cells differentiation. Sorted human naïve T cells were cultured in Th9 condition with or without 2-DG and expression of glycolytic genes were tested. Our data indicated that 2-DG significantly suppressed the expression of glycolytic genes in Th9 cells (Supplementary Figure 3). Our data further indicated that blocking of glycolysis with 2-DG suppressed ATP generation and IL-9 production (Figures 4K–M). Taken together these data indicated that ATP-HIF-1α axis is essential for the differentiation of human Th9 cells.

### NO and HIF-1α Synergistically Promote Human Th9 Cell Differentiation

**Figure 5:**
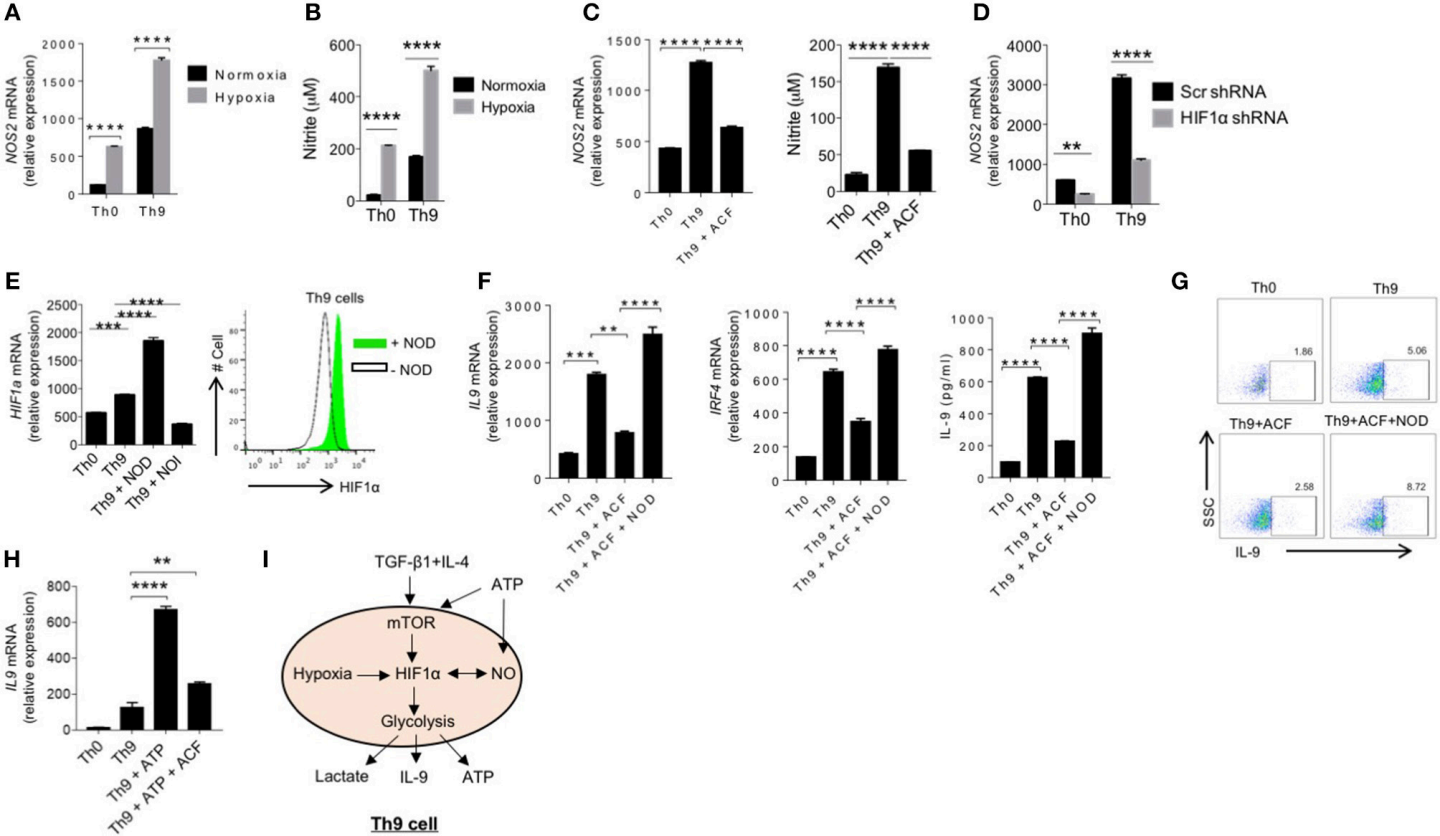
NO and HIF1α synergistically promote human Th9 cell differentiation. **(A)** Sorted naïve T cells differentiated under Th0 and Th9 polarizing conditions for 6 days in normoxia (21% oxygen) and hypoxia (1% oxygen), respectively. Total RNA was extracted, reverse transcribed and real-time PCR was done for analyzing mRNA expression of NOS2. **(B)** Nitrite measurement in Th0 and Th9 cells under normoxia and hypoxia. **(C)** Sorted naïve T cells were differentiated under Th0 and Th9 polarizing conditions for 6 days in the presence of acriflavine (ACF) followed by examination of mRNA expression of NOS2 and nitrite measurement in culture supernatants. **(D)** Sorted naïve T cells were nucleofected with naked scramble shRNA and HIF1α shRNA and were differentiated under Th0 and Th9 polarizing conditions for 6 days analyzed for mRNA expression of NOS2. **(E)** Sorted naïve T cells were differentiated under Th0 and Th9 polarizing conditions for 6 days in the presence of NOD and NOI (NOD-NO donor; NOI-NO inhibitor) followed by examination of mRNA expression of HIF1α and intracellular cytokine staining of HIF1α in the presence and absence of NOD in Th9 cells. **(F)** Sorted naïve T cells were differentiated under Th0 and Th9 polarizing conditions for 6 days alone, in the presence of ACF (Acriflavine) and NOD+ACF followed by examination of mRNA expression of IL-9 and IRF4 and IL-9 production in the culture supernatants estimated by ELISA. **(G)** Intracellular cytokine production of IL-9. **(H)** Sorted naïve T cells were differentiated under Th0 and Th9 polarizing conditions for 6 days alone, in the presence of ATP and ATP + ACF followed by examination of mRNA expression of IL-9 by qPCR. Data are representative of mean ± SEM from three independent experiments (*n* = 3). ^*^P < 0.0332, ^**^P < 0.0021, ^***^P < 0.0002, ^****^P < 0.0001; one-way ANOVA followed by Tukey's test **(C,E,F,H)**, two-way ANOVA followed by Tukey's test **(A,B,D)**. **(I)** Schematic representation of ATP mediated NO and mTOR-HIF1α signaling induces human Th9 cell differentiation.

Our data indicated that ATP-mediates NO production, which lead to further enhancement of human Th9 cells differentiation via mTOR and HIF-1α pathway. Balance between NO and free radicals, generated in ETC pathway, dictate the stability and functions of HIF-1α (40). It was found that NO stabilizes HIF-1α by making it resistant to PHD-mediated degradation (41). Our data suggest that NO is essential for the differentiation of Th9 cells, therefore we tested whether NO generated in human Th9 cells is linked to HIF-1α-mediated generation of human Th9 cells. To test our hypothesis, we tested and found Th9 cells cultured under hypoxic environment enhances IL-9 and NO production in Th9 cells (Figures 5A,B), suggesting a potential link of HIF-1α in generation of NO in Th9 cells. To directly test the role of HIF-1α in the production of NO in Th9 cells, we suppressed the HIF-1α function by acriflavine, which is known to suppress transcriptional activity of HIF-1α. Interestingly, inhibition of HIF-1α transcriptional functions significantly blocked the expression of NOS2 mRNA as well as NO production in Th9 cells (Figure 5C).

To further validate our observations, we inhibited HIF-1α using HIF-1α shRNA and determined NOS2 mRNA expression in human Th9 cells. Consistently, our data indicate that suppression of HIF-1α functions inhibited NOS2 expression in Th9 cells (Figure 5D). To further understand the association of HIF-1α and NO, we further tested whether NO modulate HIF-1α expression in human Th9 cells. To do this, we generated human Th9 cells either in the presence of NO inhibitor or NO donor to test its effect on HIF-1α expression. Our data indicated that NO donor enhanced while NO inhibitor suppressed HIF-1α expression, respectively and IL-9 induction in human Th9 cells (Figure 5E), indicating that HIF1α-NO work in feed-forward loop to promote differentiation of human Th9 cells. Similar to HIF-1α expression, NOD or NOI, respectively enhanced or suppressed the expression of Glut1 and glycolytic genes (Supplementary Figure 4). This led us to hypothesize whether HIF-1α-mediated NO production is critical for IL-9 induction and human Th9 cells differentiation, and if that is the case, whether exogenous NO can overcome the requirement of HIF-1α in human Th9 cells differentiation. To do this, we suppressed the functions of HIF-1α using acriflavine in the presence or absence of NOD. Interestingly, we found that the supplementation of exogenous NO could rescue the HIF-1α inhibition in human Th9 cells differentiation, indicating that HIF-1α-mediated generation of NO is critical for differentiation of Th9 cells (Figures 5F,G). Taken together the data suggests that HIF-1α is essential for NO-mediated human Th9 cells differentiation. To further understand whether eATP enhances IL-9 induction in Th9 cells is dependent on HIF-1α, we blocked HIF-1α functions using acriflavine (ACF) in the presence of eATP, and found that eATP-mediated enhancement of IL-9 in Th9 cells is suppressed in the presence of HIF-1α inhibitor, suggesting that HIF-1α is crucial for enhancing IL-9 by ATP (Figure 5H).

Based on our data, we proposed a schematic model of human Th9 cell differentiation, in which TGF-β and IL-4 initiate the differentiation of human Th9 cells further potentiated by ATP-mediated purinergic signaling. ATP further induces the NO production in human Th9 cells and activates inter-dependent mTOR-HIF-1α pathways and together contributes the promotion of human Th9 cell differentiation in a feed-forward loop (Figure 5I).

## Discussion

Effector T cell subsets differentiate from naïve CD4^+^ T cells in the presence of specialized cytokine milieu (42). As compared to other effector T cell subsets, IL-9-producing Th9 cells are found to be less well-characterized, though the cytokines and transcription factors that leads to the induction of human Th9 cells are identified (20, 43–45), the role of other environmental factors, such as metabolites in the differentiation of Th9 cells are yet to be identified. In this study we have shown the extracellular ATP (eATP) induces the differentiation of Th9 cells, as inhibition of purinergic receptor signaling suppressed the generation of human Th9 cells. We further demonstrated that ATP-mediated NO is essential for the differentiation of human Th9 cells, as exogenous NO could rescue the generation of human Th9 cells even in the absence of purinergic receptor signaling. Our findings further identify as to how ATP-nitric oxide potentiate mTOR-HIF-1α-mediated pathway that leads to the induction of IL-9 in human Th9 cells.

Initially, IL-9 found to be produced by activated T cells and suggested to be a T cell growth factor (1, 46). Before the identification of Th9 cells, IL-9 thought to be a Th2 cytokine, and as a result the role of IL-9 was tested in Th2-associated disease (47) models (9). Although IL-9 found to be produced by subsets of T cells, such as Th2, Th17, iTregs as well as ILCs, Th9 cells found to exclusively produce IL-9 (20, 48). IL-9 found to be associated with human conditions, such as allergy and asthma, as both IL-9 and IL-9R found to have genetic association with human asthma. Consistently, over-expression of IL-9 within the lung was found to be associated with enhanced infiltration of eosinophils and lymphocytes.

Th9 cells were found to be most potent anti-tumor T cells. Although IL-9 was found to be associated with multiple diseases, the clarity of IL-9 functions in immune responses associated with diseases came only after the identification of Th9 cells (10, 11, 49).

Activation of naïve CD4^+^ T cells in the presence of TGF-β1 and IL-4 induces the differentiation of IL-9-producing Th9 cells. Combination of TGF-β1 and IL-4 induces a distinct differentiation program as compared to TGF-β1 or IL-4 alone (10, 11). While TGF-β1 induces the generation of induced Tregs (iTregs) by inducing the expression of Foxp3, addition of IL-4 suppresses Foxp3 expression resulting the induction of IL-9-producing Th9 cells (10). Although the differentiation factors of Th9 cells was identified, other factors that enhances the differentiation of Th9 cells yet to be defined. Considering the important role of metabolic checkpoints, we identified as to how cellular ATP contributes to the differentiation of human Th9 cells. The role of ATP in regulation of T cells differentiation and functions has been identified. During the activation and differentiation of T cells, ATP is generated to fuel the T cell proliferation (29). An additional role of ATP as an extracellular signaling molecule has also been demonstrated, as it mediates cell to cell communication in an autocrine and paracrine manner. Under physiological conditions, cellular ATP is released into extracellular milieu, and therefore activates purinergic receptors signaling upon its binding to P2X and P2Y surface receptors. The role of ATP was found to be associated with Th17 cells differentiation, as ATP found to enhance the expression of TGF-β, IL-6, IL-23p19, and thus enhanced Th17 cell generation and exacerbated T-cell-mediated colitis in mouse model (23, 24). Similar to Th17 cells, we have shown here that eATP signals through purinergic receptors and enhances the human Th9 cells differentiation. However, it is not clear whether ATP controls, if at all, the generation of Foxp3^+^ Tregs and Th9 cells reciprocally, as it was established in case of Foxp3^+^ Tregs and Th17 cells (31, 50).

The role of NO was identified in differentiation and functions of Th cells, as NOS2-deficient mice were found to harbor enhanced frequency of Th17 cells with the reduction in Tregs cells in EAE, a mouse model of multiple sclerosis, indicating that NO is suppressed in Th17 cells differentiation (51). Subsequent study has identified that NO induces nitration of tyrosine residue in ROR-γt leading to the inhibition of ROR-γt-mediated induction of IL-17 in Th17 cells (52). In addition to Th17 cells, the role of NO was also identified in Th9 cells, as NO suppresses IL-17 and enhances IL-9 in Th17 cells. It was also shown that NO enhances differentiation of Th9 cells associated with lung pathologies. How NO generated during human Th9 cells differentiation is not clearly understood. Here we found that ATP-mediated NO production is essential to promote Th9 cells differentiation. We have shown that ATP-NO forms a feed-forward loop to promote IL-9 production in Th9 cells.

We further tested as to how ATP-NO modulate mTOR signaling in human Th9 cells. The role of mTOR pathways was found to be associated in Th9 cells differentiation, however, how ATP-NO axis regulate the mTOR pathway in human Th9 cells differentiation was not clearly understood. Our data indicated that eATP modulated mTOR pathway by enhancing the activation of S6 Kinase in human Th9 cells. Inhibition of mTOR suppressed both IL-9 and NOS2 induction in human Th9 cells. We further shown that ATP-mediated NO induces mTOR activation in human Th9 cells differentiation.

Since mTOR pathways leads to HIF-1α pathway, and the role of HIF-1α was suggested in the both Th9 and Th17 cells differentiation. However, it was not really known whether ATP-NO axis mediates HIF-1 alpha activation that leads to the differentiation of human Th9 cells. To connect this axis, our data indicates that ATP-induced the NO-mTOR-dependent induction of HIF-1α in human Th9 cells differentiation. Interestingly ATP and NO found to induce HIF-1α activation, which we found to further support the differentiation of human Th9 cells differentiation, as inhibition of HIF-1α leads to the inhibition of IL-9 induction in human Th9 cells.

Altogether, our work suggested that eATP enhances the differentiation of human Th9 cells and might function as a checkpoint of downstream mTOR-HIF-1α axis. Our data further emphasized the role of NO which is induced by eATP, which further potentiates glycolytic activity dependent on HIF-1α, and modulation of ATP-NO-HIF-1α axis may confer and contribute to the anti-tumor functions of Th9 cells.

## Ethics Statement

All human experiments were performed in accordance to the approved guidelines of Human Ethics Committee of THSTI. Human blood samples were collected from healthy individuals after the written informed consent. Healthy individuals were enrolled in this study based on the inclusion/exclusion criteria prescribed by the Human Ethics Committee of THSTI.

## Author Contributions

SR designed, performed the experiments, and analyzed the data. AA wrote the paper, designed, and supervised the study. SR and AA contributed to writing and editing the paper..

### Conflict of Interest Statement

The authors declare that the research was conducted in the absence of any commercial or financial relationships that could be construed as a potential conflict of interest.

## Supplementary Material

Supplementary Figure 1 Inhibition of mTOR signaling downregulates glycolysis resulting in diminished human Th9 cells differentiation. Sorted naïve T cells were differentiated under Th0 and Th9 polarizing conditions for 6 days in the absence and presence of Rapamycin (Rapa) followed by relative mRNA expression of glycolytic genes examined by qPCR. Data are representative of mean ± SEM from three independent experiments (*n* = 3). ^*^*P* < 0.0332, ^**^*P* < 0.0021, ^***^*P* < 0.0002, ^****^*P* < 0.0001; one-way ANOVA followed by Tukey's test.

Supplementary Figure 2 HIF1α is required for glycolytic activity in human Th9 cells. **(A)** Sorted naïve T cells were differentiated under Th0 and Th9 polarizing conditions for 6 days in the absence and presence of acriflavine (ACF) followed by relative mRNA expression of glycolytic genes examined by qPCR. **(B)** Sorted naïve T cells differentiated under Th0 and Th9 polarizing conditions for 6 days in normoxia (21% oxygen) and hypoxia (1% oxygen), respectively followed by mRNA expression of glycolytic genes. Data are representative of mean ± SEM from three independent experiments (*n* = 3). ^*^*P* < 0.0332, ^**^*P* < 0.0021, ^***^*P* < 0.0002, ^****^*P* < 0.0001; one-way ANOVA followed by Tukey's test **(A)**, two-way ANOVA followed by Tukey's test **(B)**.

Supplementary Figure 3 Blocking glycolysis inhibits glycolytic genes in human Th9 cells. Sorted naïve T cells were differentiated under Th0 and Th9 polarizing conditions for 6 days in the absence and presence of 2-DG followed by examination of mRNA expression profile of glycolytic genes. Data are representative of mean ± SEM from three independent experiments (*n* = 3). ^*^*P* < 0.0332, ^**^*P* < 0.0021, ^***^*P* < 0.0002, ^****^*P* < 0.0001; one-way ANOVA followed by Tukey's test.

Supplementary Figure 4 Nitric oxide (NO) is crucial for enhanced glycolysis in human Th9 cells. Sorted naïve T cells were differentiated under Th0 and Th9 polarizing conditions for 6 days in the absence and presence of 2-DG followed by examination of mRNA expression profile of glycolytic genes. Data are representative of mean ± SEM from three independent experiments (*n* = 3). ^*^*P* < 0.0332, ^**^*P* < 0.0021, ^***^*P* < 0.0002, ^****^*P* < 0.0001; one-way ANOVA followed by Tukey's test.
