# Supplementary figures and images for: ATP Triggers Human Th9 Cell Differentiation via Nitric Oxide-Mediated mTOR-HIF1α Pathway
Source: Front Immunol. 2019 May 20;10:1120. doi: 10.3389/fimmu.2019.01120 (PMC6536008; doi:10.3389/fimmu.2019.01120)

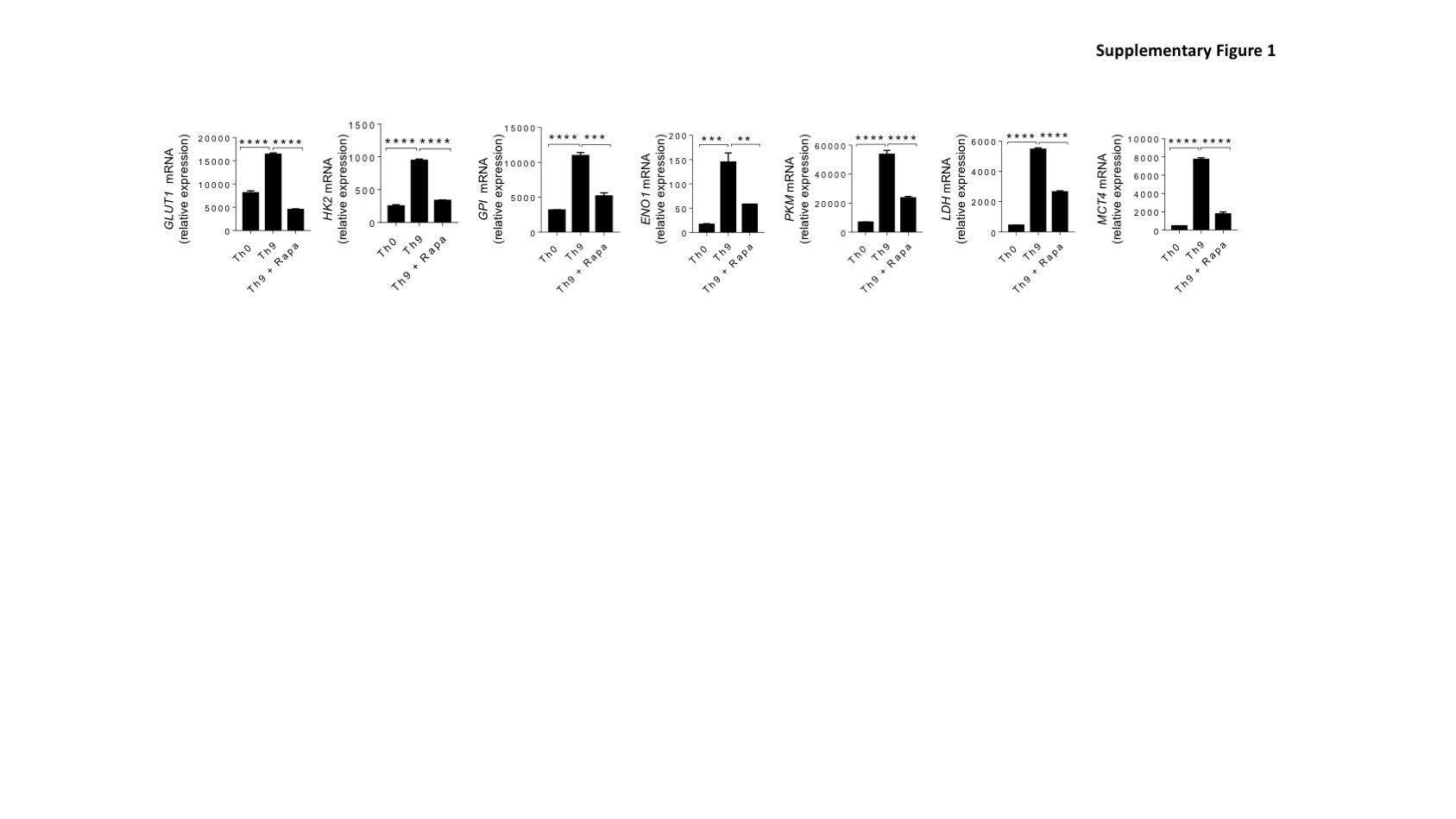

Supplement: Supplementary Figure 1 — Inhibition of mTOR signaling downregulates glycolysis resulting in diminished human Th9 cells differentiation. Sorted naïve T cells were differentiated under Th0 and Th9 polarizing conditions for 6 days in the absence and presence of Rapamycin (Rapa) followed by relative mRNA expression of glycolytic genes examined by qPCR. Data are representative of mean ± SEM from three independent experiments (n = 3). *P < 0.0332, **P < 0.0021, ***P < 0.0002, ****P < 0.0001; one-way ANOVA followed by Tukey's test. [file Image_1.jpeg]

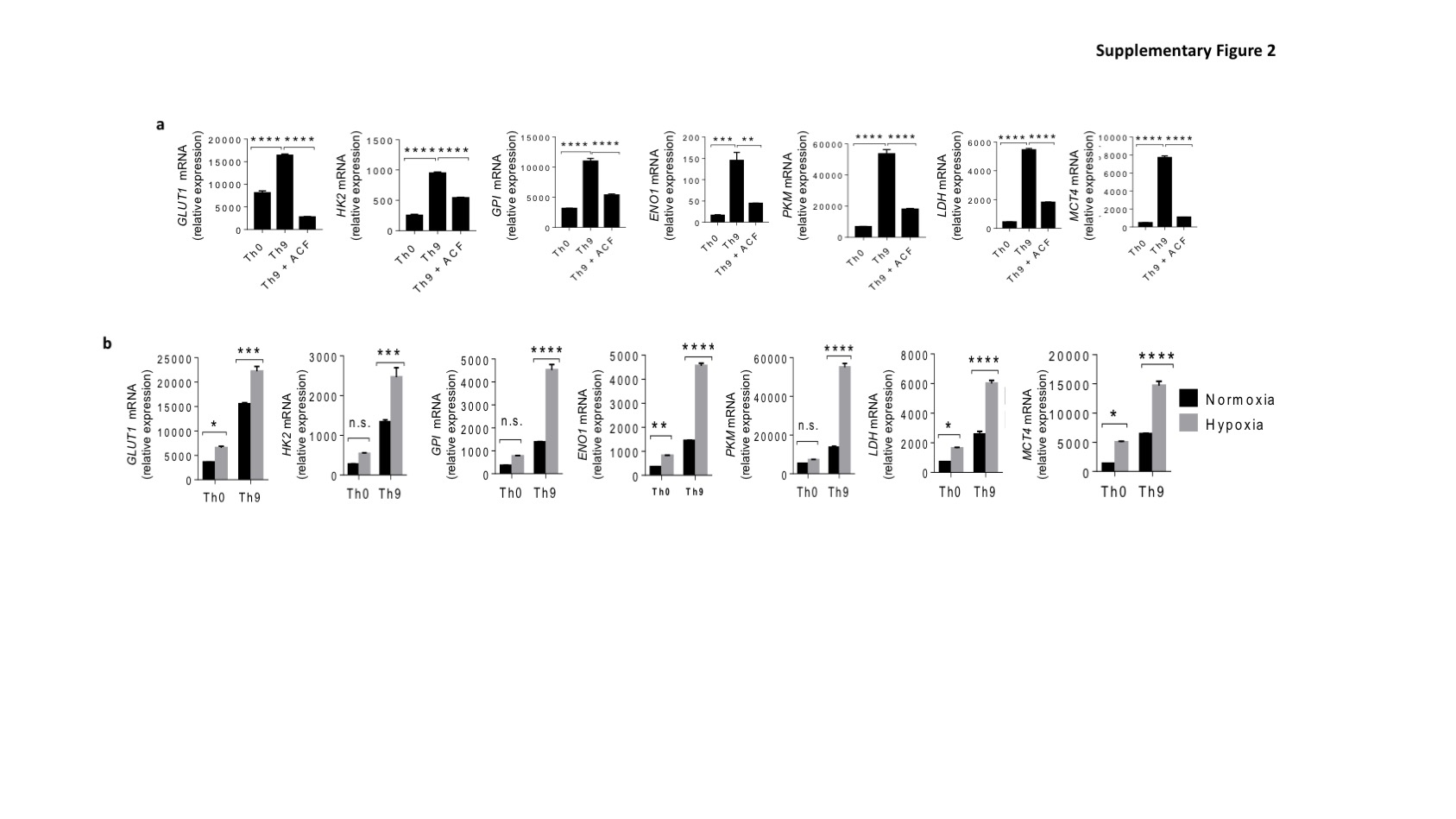

Supplement: Supplementary Figure 2 — HIF1α is required for glycolytic activity in human Th9 cells. (A) Sorted naïve T cells were differentiated under Th0 and Th9 polarizing conditions for 6 days in the absence and presence of acriflavine (ACF) followed by relative mRNA expression of glycolytic genes examined by qPCR. (B) Sorted naïve T cells differentiated under Th0 and Th9 polarizing conditions for 6 days in normoxia (21% oxygen) and hypoxia (1% oxygen), respectively followed by mRNA expression of glycolytic genes. Data are representative of mean ± SEM from three independent experiments (n = 3). *P < 0.0332, **P < 0.0021, ***P < 0.0002, ****P < 0.0001; one-way ANOVA followed by Tukey's test (A), two-way ANOVA followed by Tukey's test (B). [file Image_2.jpeg]

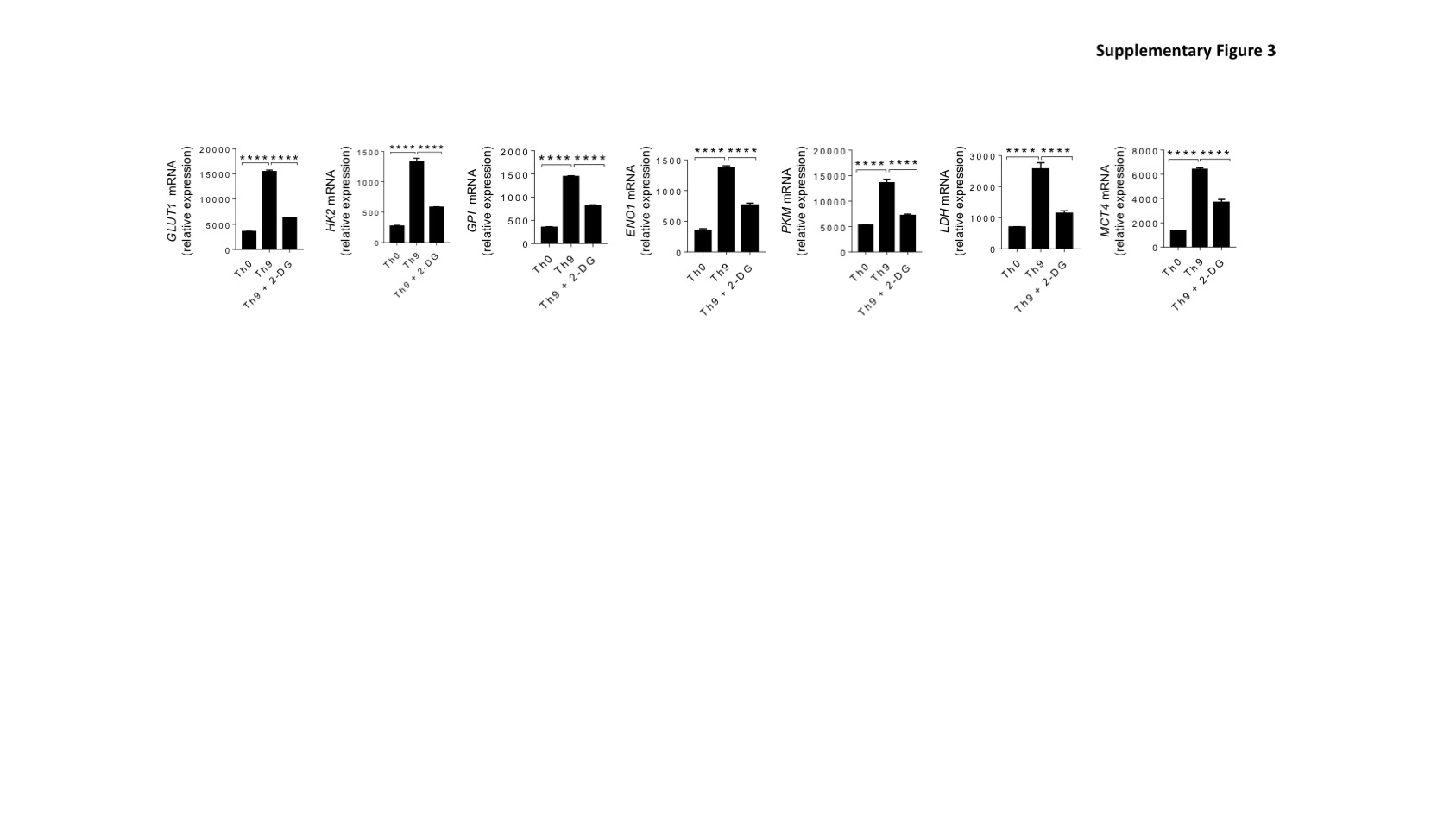

Supplement: Supplementary Figure 3 — Blocking glycolysis inhibits glycolytic genes in human Th9 cells. Sorted naïve T cells were differentiated under Th0 and Th9 polarizing conditions for 6 days in the absence and presence of 2-DG followed by examination of mRNA expression profile of glycolytic genes. Data are representative of mean ± SEM from three independent experiments (n = 3). *P < 0.0332, **P < 0.0021, ***P < 0.0002, ****P < 0.0001; one-way ANOVA followed by Tukey's test. [file Image_3.jpeg]

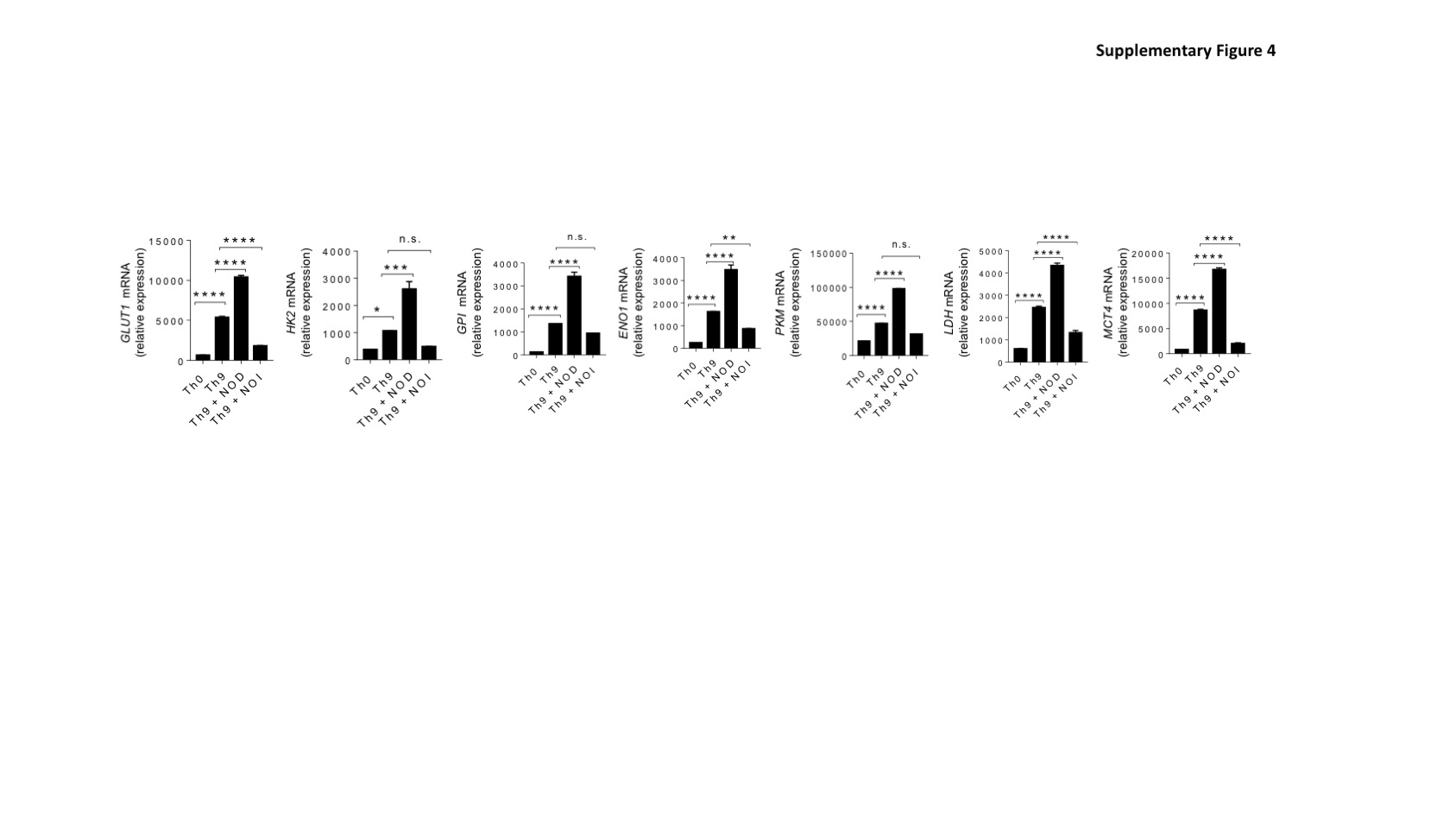

Supplement: Supplementary Figure 4 — Nitric oxide (NO) is crucial for enhanced glycolysis in human Th9 cells. Sorted naïve T cells were differentiated under Th0 and Th9 polarizing conditions for 6 days in the absence and presence of 2-DG followed by examination of mRNA expression profile of glycolytic genes. Data are representative of mean ± SEM from three independent experiments (n = 3). *P < 0.0332, **P < 0.0021, ***P < 0.0002, ****P < 0.0001; one-way ANOVA followed by Tukey's test. [file Image_4.jpeg]
